# Parent-identified intrinsic and extrinsic factors that influence performance across developmental domains and participation in their communities

**DOI:** 10.3389/fped.2025.1472743

**Published:** 2025-02-24

**Authors:** Rachel Bican, Sydney Shaffer, Jayna Kinkade, Quinn McAdams, Allyson S. Hughes

**Affiliations:** ^1^ Department of Physical Therapy, College of Health Sciences and Professions, Ohio University, Athens, OH, United States; ^2^ Department of Primary Care, Ohio University Heritage College of Osteopathic Medicine, Athens, OH, United States

**Keywords:** autism spectrum disorder (ASD), attention deficit hyperactivity disorder (ADHD), developmental disabilities, rural health, Appalachia

## Abstract

Children with neurodevelopmental disabilities living in rural and low-resourced regions within the United States, such as Appalachia, face gaps and barriers to accessing healthcare services due to a shortage of providers, specialists, hospitals, and clinics. Without access to specialized medical and rehabilitation services, their performance across developmental domains and participation within their communities is likely suboptimal. The purpose of this study was to identify both intrinsic and extrinsic factors using a mixed-methods approach to better understand factors that may impact performance across developmental domains and participation for children with disabilities living in Appalachia. Parents completed one study visit in which they completed a total of 4 surveys and a semi-structured interview. The surveys included a parent survey (demographic information, medical history for the child, and barriers to receiving healthcare for their child), health literacy screen, the F-Words Life Wheel, and the Pediatric Evaluation of Disability Inventory (PEDI-CAT). The semi-structured interview asked questions in five primary categories: (1) background, (2) understanding of their child's medical diagnosis and management of their disability, (3) insurance coverage, (4) barriers to receiving healthcare, and (5) social support. This cross-sectional study included *n* = 17 parents of *n* = 26 children with neurodevelopmental disabilities. Themes from the interviews were coded both inductively and deductively. Most of the children had delays in important developmental domains, indicating a need for rehabilitation services. Participants reported significant difficulty finding specialists due to the distance from their house to the specialist, they experienced long waitlists and delayed diagnoses, they had difficulties finding caregivers for their children, they frequently had multiple children with disabilities, and they experience sleep disruptions due to their child(ren) with neurodevelopmental disabilities. The authors provide clinical research recommendations and policy changes that may be considered to help mitigate barriers to healthcare for children with neurodevelopmental disabilities living in rural and low-resourced regions.

## 1 Introduction

Children born in Appalachia, a rural and low-resourced region, face tremendous health inequities when compared to the rest of the United States (US) population (1, 2). Appalachia is a socioeconomically disadvantaged geographical and cultural region in the eastern US (2). Infants born in Appalachia are at a higher risk for being born prematurely or at a low birthweight (2–4) and children in Appalachia have a higher prevalence of developmental delays when compared to the rest of the US (28% in Appalachia, 17% across the US) (5). These children often require comprehensive healthcare for optimal health outcomes and to promote full participation within their communities. Unfortunately, children living in Appalachia face both gaps in healthcare and barriers to accessing healthcare.

A majority of the counties in Appalachia have a federal designation as a health professional shortage area (HPSA) (6, 7). This shortage in healthcare providers and healthcare facilities results in gaps in care for primary and specialized services for both children and adults (4, 6–16). A study by Morrone et al. in 2021 (10) surveyed 695 people from both Appalachian and non-Appalachian counties and found that only 29% of survey respondents from Appalachia felt that there were enough healthcare services within their county. This was 28% lower than respondents from non-Appalachian counties (10). In addition to limited available medical resources, adults and children living in Appalachia face barriers to accessing healthcare and adhering to medical recommendations. High unemployment rates and the low socioeconomic status of individuals living in this region (2, 15, 17) make it difficult for individuals to travel to and pay for required healthcare services (8, 13–15). Many studies have cited health insurance coverage as a barrier for accessing care (4, 10, 11, 15, 17, 18) and the lack of healthcare coverage has been identified as a barrier for mothers seeking prenatal healthcare (13). Individuals living in Appalachia also have lower education and health literacy levels (2, 7, 14, 14, 18), as well as a documented distrust in the medical system (11, 15, 18), which may result in lower rates of seeking out healthcare and adherence to medical interventions.

Children with disabilities living in rural regions have higher unmet medical needs when compared to children from urban regions (5, 17). Neurodevelopmental disabilities, such as attention deficit disorder (ADD), attention deficit hyperactivity disorder (ADHD), and autism spectrum disorder (ASD), often result in delays across developmental domains, including cognitive, learning, motor, speech and language, and social emotional skills (19–21). To address these delays, children with neurodevelopmental disabilities require comprehensive interventions from medical providers, including rehabilitation specialists, for optimal participation within their communities (19, 20, 22). There is a single study by Scarpa et al. (23) that specifically investigates gaps in healthcare and barriers individuals with ASD face living in Appalachia. For this study, 15 caregivers of children with ASD and 33 service providers were included. They report limited availability and lack of affordability of ASD services. They also report limited availability of rehabilitation specialists, including physical, occupational, and speech therapists (23). The lack of specialty providers, and subsequent lack of specialty interventions, likely impacts a child's performance and participation within their communities, however, the relationship between these factors has not yet been studied.

The purpose of our study was to describe factors that may influence performance across developmental domains and participation for children with neurodevelopmental disabilities living in Appalachia, which may help to inform the development of targeted clinical interventions. To do this, we utilized the Person-Environment-Occupation-Performance (PEOP) Framework. The PEOP was first proposed by Law et al. (24) in 1996 as a theoretical model that suggests that performance and participation within their social roles are influenced by the person, environment, and occupational activities or tasks. This top-down, systems model was later updated by Baum et al. in 2015 (25, 26). This updated PEOP suggests that performance and participation are influenced by both intrinsic (personal) factors and extrinsic (environmental) factors (24, 25). The study described in this manuscript seeks to identify both intrinsic and extrinsic factors using a mixed-methods approach to better understand factors that may impact performance across developmental domains and participation for children with disabilities living in Appalachia. The authors will also discuss opportunities for clinical research and policy changes to improve the health and wellbeing of children with neurodevelopmental disabilities living in Appalachia, which may be relevant to other rural and low-resourced communities.

## 2 Materials and methods

### 2.1 Participants

The procedures described in this study were approved by the Institutional Review Board at Ohio University (IRB-FY24-129). Informed written consent was obtained from all participants. The primary aim of the larger study was to evaluate the relationship between intrinsic/extrinsic factors and performance across developmental domains and participation for children with developmental delays living in Appalachia.

This study included a subset of the participants from the larger study. The subset of participants for this manuscript was determined by the following inclusion and exclusion criteria. Inclusion criteria: Parents of children (1) ages 0–17 years old, (2) living in an Appalachian County >50% of the time, and (3) has a neurodevelopmental disability including ADD, ADHD, ASD, or are pending a diagnosis for a neurodevelopmental disability. Exclusion criteria: (1) non-English speaking. Although ADD, ADHD, and ASD range in etiology and severity, we chose to include all diagnoses in this study due to similar parent-reported challenges/impairments across developmental domains and behaviors that these children experience. This sample size allowed for saturation of themes to be identified from the semi-structured interviews. The primary aim of this study is to describe intrinsic and extrinsic factors that may impact performance across developmental domains and participation based on the PEOP Framework.

### 2.2 Study procedures

Participants completed one study visit that took between 2.5 and 3 h to complete. Study visits took place at the participant's home, at Ohio University, or online via Microsoft Teams. Parent participants chose the location of the study visit based on convenience and comfortability.

#### 2.2.1 Outcomes

Parents completed a total of 4 surveys and 1 semi-structured interview. The surveys and the semi-structured interview collect data on intrinsic factors (factors related to the child and family), extrinsic factors (factors related to the child's home and community environment), performance across developmental domains, and participation within the home and community.

##### 2.2.1.1 Parent survey

During the study visit, participants completed an initial parent survey that collected both intrinsic and extrinsic factor information. The survey included demographics, medical history of the child, rehabilitation use, and barriers to accessing healthcare. The survey included open response and selection from options (see Supplementary Material for the full survey). This survey took about 15 min for the participant to complete. A researcher was available to answer any questions from the parent participant while they were completing the survey.

##### 2.2.1.2 Health literacy screen

Participants completed the Rapid Estimate of Adult Literacy in Medicine-Short Form (REALM-SF) (26) as part of the intrinsic factors. This screen is a 7-item word recognition test and is a validated instrument for assessing participant health literacy. Participants were handed the REALM-SF and were asked to read the words out loud and that if they were unable to identify the word, they could say pass. The words include behavior, exercise, menopause, rectal, antibiotics, anemia, and jaundice. Participants receive 1-point for every correct word for a total of 7-points. The points on the screen can be used to determine a grade-level literacy score ranging from below third grade level to high school level.

##### 2.2.1.3 Performance across developmental domains and participation outcomes

Participants completed the F-Words Life Wheel. The F-Words Life Wheel provides families with a framework to discuss goals for their child across key developmental domains and is based on the International Classification of Functioning, Disability, and Health (27). These domains include the following: (1) family, (2) function in the home/community, (3) fitness, (4) fun, (5) friends, and (6) future. Specifically, the domains of Function and Fun are associated with performance and participation. Participants were handed the F-Words Life Wheel handout and were read a blurb for each of the domains by the researcher. The researcher then asked them to circle a score from 1 (least satisfied) to 10 (most satisfied) regarding their child's current function within the domain. The F-Words Life Wheel is available for free from CANChild.

Participants completed the Pediatric Evaluation of Disability Inventory, Computerized Adapted Test (PEDI-CAT). The PEDI-CAT is a responsive (*r* = 0.78) (28) and reliable (ICC = 0.96) assessment for children ages 0–12 years old to capture performance and participation in four domains: Daily Activities, Mobility, Social/Cognitive, and Responsibility (29, 30).

##### 2.2.1.4 Semi-structured interviews

The participants also completed a semi-structured interview facilitated by one trained researcher (See Supplementary Material for the full interview guide). The interviews were video and audio recorded. The interview guide was developed by the researchers on the team based on the PEOP and on previous research. The interview questions spanned both intrinsic and extrinsic factors that may influence performance and participation for children with neurodevelopmental disabilities living in Appalachia. Five main categories were identified to serve as a starting point for the development of the interview questions. These categories included (1) the family and child's background, including medical history, (2) the participant's understanding of their child's medical diagnosis and management of their disability, (3) insurance coverage in regards to accessing healthcare, (4) barriers to receiving healthcare, including availability of primary and specialty care, transportation, and travel, and (5) perceived social support from the medical and educational systems. Table 1 presents examples of questions in each category. Interviews took approximately 60 min to complete and were video and audio recorded.

**Table 1:** Examples of questions on the semi-structured interview.

Category	Questions
Background	*How would you describe the area you live in?* *Are you currently working?* *What medical conditions does your child have?*
Understanding of their child's medical diagnosis and management of their disability	*How long ago was your child diagnosed with [conditions]?* *How do you work with your child to manage their health?*
Insurance coverage	*What are the pros and cons of your health insurance coverage?* *From a parent's perspective, is there anything you would do to improve your child's health insurance?* *Do you feel you are easily able to find a doctor or specialist to meet the needs of your child?*
Barriers to receiving healthcare	*Have you ever experienced barriers to care due to your distance from the hospital or other health clinics?* *How far are you typically driving for an appointment?* *Do you feel like the care your child receives is adequate?*
Social support	*Where do you receive social support and from whom?* *What do teachers do to help manage your child's welfare?* *Does your child's coaches or gym teachers make accommodations for your child?*

### 2.3 Statistical analysis

Descriptive statistics (means, standard deviations, ranges, percentages) were used to report the findings from the parent participant survey, REALM-SF, F-Words Life Wheel, and the PEDI-CAT. Qualitative data analysis was completed to develop themes from the semi-structured interviews. Interviews were video and audio recorded and were transcribed verbatim. A codebook was developed by the lead qualitative research on this study (AH). Themes were determined both inductively and deductively using the latest version of NVivo and following a foundation of thematic analysis (31). Two coders (AH and SS) separately coded the interviews to determine themes and appropriate quotes. Coders then met to discuss analysis and reach consensus. Cohen's kappa was calculated to measure interrater reliability across both coders and showed substantial agreement across both coders (kappa = .78).

## 3 Results

### 3.1 Parent survey

For this cross-sectional study, *n* = 17 parents of *n* = 26 children with a neurodevelopmental disability participated. This study included a subset of participants from a larger study that included *n* *=* 29 parents of *n* *=* 40 children with developmental delays. Inclusion criteria for larger study included: Parents of children (1) ages 0–17 years old, (2) living in an Appalachian County >50% of the time, and (3) has a diagnosed developmental delay. Of the parent participants, *n* = 16 are mothers and *n* = 1 is a father. Demographic information including parent and child age, sex, race, and ethnicity; and parent education level, marital status, employment, and income are presented in Table 2.

**Table 2:** Parent and child demographic information.

Parent participant	Mean ± SD (Range)
Age (years)	37.5 ± 6.9 (25.0–47.0)
	*N* (%)
Sex (male)	1 (5.9)
Race	
Asian	1 (5.9)
White	16 (94.1)
Ethnicity	
Hispanic	1 (5.9)
Not Hispanic	16 (94.1)
Education	
Did not complete high school	1 (5.9)
High school diploma or GED	6 (35.3)
Vocational or Associate's degree	1 (5.9)
Bachelor's degree	2 (11.8)
Master's degree	5 (29.3)
Doctoral degree	2 (11.8)
Marital status	
Not married	1 (5.9)
Separated or divorced	1 (5.9)
Married	15 (88.2)
Employment	
No	9 (52.9)
Yes, part-time	2 (11.8)
Yes, full-time	6 (35.3)
Spouse/partner employment	
No spouse or partner	1 (5.9)
No	3 (17.6)
Yes, part-time	2 (11.8)
Yes, full-time	11 (64.7)
Income (annual)	
<$50,000	6 (35.3)
$50,000-$74,999	4 (23.5)
$75,000-$99,999	3 (17.6)
$100,000-$149,999	2 (11.8)
$150,000-$199,000	1 (5.9)
>$200,000	1 (5.9)
Child	Mean ± SD (Range)
Age (years)	9.4 ± 4.3 (3.2–17.1)
	*N* (%)
Sex (male)	17 (65.4%)
Race	
Asian	1 (3.8)
Biracial or multiracial	1 (3.8)
White	24 (92.4)
Ethnicity	
Hispanic	1 (3.8)
Not Hispanic	25 (96.2)
Health insurance coverage	
Medicaid	10 (38.4)
Private	8 (30.7)
Both	6 (23.1)
Other	2 (7.8)

Most of the children of the participants had a diagnosis of or were suspected to have ASD (73.1%). Most children also had two or more formal medical diagnoses (80.8%). During the interviews, most parents expressed that their children had difficulty making meaningful friendships in school and in the community due to their medical diagnoses and developmental delays. The mean age of diagnosis for their child's neurodevelopmental disorder was when the child was 4.3 years old, although the mean age the parents suspected a problem was when their child was 2.3 years old. Most parents reported that they believed that the diagnosis was late (65.4%). See Table 3 for more information on the child's diagnosis and medical history.

**Table 3:** Medical history of the children with neurodevelopmental disabilities whose parents were enrolled in the study.

Child	*N* (%)
Study qualifying medical diagnosis	
ADHD	6 (23.1)
ASD	12 (46.2)
ADHD and ASD	1 (3.8)
Pending neurodevelopmental diagnosis, suspected ADHD or ASD	7 (26.9)
Other medical diagnoses	
Anxiety	5 (19.2)
Depression	1 (3.8)
Epilepsy/seizures	2 (7.7)
Global developmental delay	3 (11.5)
Obsessive compulsive disorder	1 (3.8)
Oppositional defiant disorder	1 (3.8)
Rett syndrome	1 (3.8)
Sensory processing disorder	4 (15.4)
Parent reported delays or disabilities (domains)	
Cognitive	7 (26.9)
Learning	7 (26.9)
Motor	8 (30.8)
Speech/language	13 (50.0)
Social/emotional	6 (23.1)
	Mean ± SD (Range)
Age of child at diagnosis of neurodevelopmental diagnosis (years)	4.3 ± 2.7 (1.5–15.0)
Age of the child when the parent suspected a problem/delay	2.3 ± 1.9 (0.3–7.0)
	*N* (%)
Parent perceives the diagnosis was late	
Yes	17 (65.4)
No	8 (30.8)
Not sure	1 (3.8)

Rehabilitation use is presented in Table 4. The largest proportion of children were receiving rehabilitation services once a year or less (behavioral therapy 34.7%; occupational therapy 57.7%; physical therapy 61.6%, and speech therapy 34.7%). Most parents believed that this frequency was appropriate for their child (frequency is appropriate for behavioral therapy 77.0%; occupational therapy 73.1%; physical therapy 92.3%, and speech therapy 80.8%).

**Table 4:** Rehabilitation use for the children of the parents included in the study.

Service	*N* (%)
Total children	26 (100)
Behavioral therapy	Number of children receiving this therapy (%)
Not receiving services	2 (7.7)
Once a year or less	9 (34.7)
Once a month	3 (11.5)
Every other week	2 (7.7)
Once a week	7 (26.9)
More than once a week	3 (11.5)
Occupational therapy	Number of children receiving this therapy (%)
Not receiving services	4 (15.4)
Once a year or less	15 (57.7)
Once a month	0 (0.0)
Every other week	0 (0.0)
Once a week	4 (15.4)
More than once a week	3 (11.5)
Physical therapy	Number of children receiving this therapy (%)
Not receiving services	7 (26.9)
Once a year or less	16 (61.6)
Once a month	0 (0.0)
Every other week	0 (0.0)
Once a week	2 (7.7)
More than once a week	1 (3.8)
Speech therapy	Number of children receiving this therapy (%)
Not receiving services	4 (15.4)
Once a year or less	9 (34.7)
Once a month	0 (0.0)
Every other week	0 (0.0)
Once a week	7 (26.9)
More than once a week	6 (23.0)
Parent perception of frequency of services	
Behavioral therapy	
Parent believes their child should be going less	0 (0)
Parent believes the frequency is appropriate	6 (23.0)
Parent believes their child should be going more	20 (77.0)
Occupational therapy	
Parent believes their child should be going less	0 (0.0)
Parent believes the frequency is appropriate	19 (73.1)
Parent believes their child should be going more	7 (26.9)
Physical therapy	
Parent believes their child should be going less	0 (0)
Parent believes the frequency is appropriate	24 (92.3)
Parent believes their child should be going more	2 (7.7)
Speech therapy	
Parent believes their child should be going less	0 (0.0)
Parent believes the frequency is appropriate	21 (80.8)
Parent believes their child should be going more	5 (19.2)

Most of the participants selected cost of gas (65.4%), distance to the clinic or hospital (65.4%), inability to find childcare for siblings (73.1%), insurance coverage (53.8%), lack of social support (57.7%), and personal or family schedules (88.5%) as barriers to care (Table 5). Parents were also able to write in other barriers to care. One parent reported that “*insurance deciding what meds we can try and what therapies are ‘worth it’*” was a barrier to care. Another parent reported a “*lack of understanding*” demonstrated by medical providers.

**Table 5:** Parent-reported barriers to healthcare for their child.

Barrier	*N* (%)
Cost of gas	17 (65.4)
Distance from the clinic/hospital	17 (65.4)
Hours of operation of facilities	13 (50)
Inability to find childcare for siblings	19 (73.1)
Insurance coverage	14 (53.8)
Lack of equipment	5 (19.2)
Lack of reliable transportation	9 (34.6)
Lack of social support	15 (57.7)
Personal or family work schedule	23 (88.5)
Time it takes to receive packages to arrive (containing medications or equipment)	8 (30.8)

### 3.2 Health literacy screen

All participants scored a 7/7 on the REALM-SF, indicating all were at a high school level.

### 3.3 Performance across developmental domains and participation

F-Words Life Wheel: Participant averages for each domain are presented: Family: 8.8 ± 1.2, Function at home: 7.0 ± 1.7, Function in the community: 6.7 ± 2.4, Fitness: 8.0 ± 2.0, Fun: 8.3 ± 1.4, Friends: 5.3 ± 2.7, and Future: 7.7 ± 1.7.

PEDI-CAT: Participant scores for each category are presented. Daily Activities: Scaled score = 56.3 [standard error (SE) = 0.70] (65.4% falling under the 25th percentile); Mobility: Scaled score = 68.1 (SE = 1.01) (34.6% falling under the 25th percentile); Social/Cognitive: Scaled score = 64.3 (SE = 0.77) (76.9% falling under the 25th percentile); Responsibility: Scaled score = 45.8 (SE = 1.13) (57.7% falling under the 25th percentile).

### 3.4 Semi-structured interview themes

Participants report significant difficulty finding specialists due to distance, experience long waitlists and delayed diagnoses, challenges finding caregivers for their children requiring them to stay home to be the primary caregiver, frequently have multiple children with disabilities, and experiencing sleep disruptions for them and their child(ren) with neurodevelopmental disabilities.

#### 3.4.1 Theme 1—difficulty finding specialists due to distance

Participants noted that Appalachian Ohio lacks specialists their children need for healthcare. The lack of specialists means families need to travel to larger cities in the state to receive healthcare from pediatric specialists. Due to participants' locations—participants often experience over an hour-long car ride from the Columbus and Cincinnati based specialists one way.

One participant noted the significant driving time that comes with seeing specialists by stating, “*And we tried to get in with [children's hospital 2]; to transfer our care from his care team from [children's hospital 1] to [children's hospital 2] just because it's closer. I don’t want to drive two and a half hours to go to a visit. And that was impossible*.”

Another stated: “*Specialists are not here. Specialists, you have to go to Columbus [Ohio, about a 1.5-h drive from their home]*.”

One participant said, “*I more worry because I don’t know where else to find it. So, if we lived in Columbus, I am sure if we were told by the [clinic] to leave, we probably can find somewhere else. But here, we just don’t have much options. So, I’ve been thinking of driving to Marietta [Ohio, about a 45-min drive from their home].*”

#### 3.4.2 Theme 2—waitlist and delayed diagnosis

Participants were asked what their barriers to healthcare for their children were and many expressed the issues with long waitlists to see pediatric specialists. Specialist waitlists are a common phenomenon of the US healthcare system, but early intervention is critical for developmental delays (7, 17). Importantly, waitlists may mean that their children had delayed diagnoses because they were unable to see the specialists. Participants shared that waitlists such as these can have ramifications for Individualized Educations Plans (IEPs), 504 plans, and health outcomes. Participants stated their children were waitlisted for ASD and ADHD evaluations, their children waited over a year to get into the dentist, and waited between 6 months and a year to see a specialist at the closest children's medical center which is about a 1.5-h drive from most of the participants' homes.

One participant stated: “*We first got on the waitlist at [a children's hospital], because the doctor referred us. It takes forever.*”

Another stated: “*She had been on the waitlist for almost exactly a year*”.

One participant mentioned waitlists and the lack of specialists: “*And so he couldn’t get in until July [for speech and language pathology]. So again, that stunk. But yeah, so there was only one speech therapist, and then there was no other assistant.*”

#### 3.4.3 Theme 3—caregiving and staying at home

Participants expressed a range of caregiving responses. Some stated that they had support from family or support professionals: “*We have direct support professionals that come in our house after school and help out until right around bedtime*”. To get this support (an aide at home), the interviewee stated they had to document that the child was a danger to themselves/others in the household (especially other children in the house). This is when they were asking about support at home. This child also has a one-on-one at school when asked about support there.

One participant stated that they do not want to place the burden of caregiving for their child on others, “*He has stayed the night with my in-laws before but I always.. I hate doing it because I know that that means they’re just not getting any sleep. Like my mother-in-law is just not going to sleep that night. She's in her 60s. She's got her own medical issues. She needs to sleep. So, I just don’t like putting her through that.*”

Another family has strong support from the county: “*We have the same [medical] teams for both kids, and they actually have the same [social] workers mostly through the Family and Children First Council, County Board of Duty, all of that. At least as of right now, they have the same [social] worker. There's been times where we’ve had different [social] workers, and it's just easier to have the same [social] worker*.”

Several participants stated that they cannot find caregivers and that it produces a lot of anxiety for them. Many participants said they were unable to work due to taking care of their children with disabilities. Participants expressed uncertainty: “*I don’t know what they [in reference to a babysitter or caregiver] will do with kids who will have a meltdown. I mean even they [caregiver] are full well.. they are very well intentioned; I just don’t know.. I mean whether they [caregiver] have the skills or experience.*”

Another stated: “*… I don’t know if he would handle somebody [in reference to a babysitter or caregiver], if he [the child] had a physical outburst, if they [the caregiver] would be able to handle it or if they [the caregiver] would hurt him or if he would hurt them. Or if they [caregiver] can’t handle him, would they involve law enforcement or something? I’m more worried about the physical outbursts that he [the child] has*.”

#### 3.4.4 Theme 4—multiple children with disabilities

Seven of the participants interviewed have multiple children with disabilities, and interviewees described the challenges faced when caring for them. One participant stated, “*Well, and I think maybe it would have been better if he was my only child or if I’d had a different older child [who also has a disability], but because I was dealing with [older child]…who had severe behavioral issues that whole year.*”

Another expressed how they felt when their second child received their diagnosis: “*Horrible. You know it. You know it's coming, because you’ve already been down this path. You know. In that respect, it was horrible, because you know this is what it's going to feel like again. But on another end, you know so much more about what to do [in regards to getting a diagnosis for the second child].*”

One participant brought up the different sensory challenges their children face: “*And having kids with sensory issues with the food makes it even harder, because [child 1] doesn’t eat pasta. And both the kids are on different ends of the spectrum. [Child 2] is a seeker, [child 1] is an avoider.. But everybody's like, how come you eat two meals for dinner every night? And I’m like, because if three out of four of us eat it, it's a good night. Most of the time, I make two meals and two people are going to eat it and two people are going to eat the other meal.*”

#### 3.4.5 Theme 5—sleep dysfunction

Several participants stated there is significant sleep disruption with their child(ren) with disability and that it also impacts their sleep.

“He [the child] would rather wake up at 3:00 or 4:00 in the afternoon, do his tutoring, wake up completely, and then get something to eat. And then he’ll [the child] come back at like midnight, and I’ll be like, “Okay, I need you to tell me so many facts about whatever.” And he’ll come back and be like, “I watched this really cool documentary on this. Can I study this tonight?” And I'm like, “Yeah, knock yourself silly.” [..] we'll be having a conversation at three o'clock in the morning about the Roman Empire or how the Canadians burned down the White House or whatever.”

Another stated: “*Definitely do feel like it would be a burden particularly at night because he [the child] does not sleep. He just doesn’t sleep.*”

One participant has frequent sleep disruptions due to their child: “*waking up, yes. I will literally wake up in the middle of the night and hear something and look at the foot of my bed, and he's [the child] just kind of standing there.*”

## 4 Discussion

Parents of children with neurodevelopmental disabilities living in Appalachia face immense difficulties maintaining the health and well-being of their children. Children with disabilities often require comprehensive medical and rehabilitation services to promote performance across developmental domains and participation within their communities. Medically underserved regions of the US, such as Appalachia, have gaps in available healthcare and parents of children with disabilities face many barriers when trying to access healthcare. This likely negatively impacts a child's developmental skills and their ability to fully participate in their communities. The aim of the study is to identify intrinsic (personal) and extrinsic (environmental) factors that may impact performance across developmental domains and participation.

Parents reported that their children had delays in important performance and participation domains. Our study found that ∼34% of the children in this study fell below the 25th percentile in Mobility domain and ∼77% of children fell below the 25th percentile in Social/Cognitive domain on the PEDI-CAT. Parent satisfaction with their child's functioning within the home and community averaged 6.9 out of 10 on the F-Words Life Wheel. These findings would suggest that most of the children would benefit from rehabilitation services and targeted interventions. However, our study found significant gaps in healthcare for local specialists, resulting in many barriers to accessing the care their child needs.

Parents reported many barriers to accessing and adhering to medical and rehabilitation care related both to intrinsic and extrinsic factors. Two major themes were identified as barriers to care during the semi-structured interviews: (1) distance from the hospital or clinic, and (2) waitlist and delayed diagnoses. Most parents in the study must drive to the nearest children's hospital, about 1.5 h away, to get specialized care for their child. Some families reported making this drive several times a month. To make these appointments, it requires the parent and family to have flexibility in their schedule (88.5% reported as a barrier), money to pay for gas (65.4% reported as a barrier), care for other siblings if they are unable to attend the appointment (73.1% reported as a barrier), and reliable transportation (34.6% reported as a barrier). The magnitude of the effort the parent must put in to adhere to these medical appointments becomes clear once all barriers are accounted for.

Parents also reported difficulty scheduling an appointment due to long waitlists at specialized clinics to receive a neurodevelopmental diagnosis for their child. Approximately 65.4% of the parents perceived that their child's diagnosis was delayed. Parents, on average, identified a “problem” when their child was 2.3 years old; however, parents reported the medical diagnosis was not given on average until their child was 4.3 years old. Waitlists to get into these specialized clinics in this region are roughly 1 year, although early diagnosis and subsequent intervention are critical for best outcomes for children with neurodevelopmental disabilities (7, 17). Due to the lack of service providers, there are also waitlists for rehabilitation providers. Parents report that when they start rehabilitation, the frequency of services can be very low. The largest proportion of children report being seen once a year or less. This is particularly worrisome because children with neurodevelopmental disabilities likely need rehabilitation; however, accessing these services more frequently for education, evaluation, and treatment may be difficult.

Our study examined several intrinsic factors including socioeconomic components, health literacy, and the child's medical history that may impact performance and participation. When examining socioeconomic factors of the family, most parents (58.8%) reported having additional training after high school or a college level degree, yet most were not currently employed (52.9%). Many parents reported that their unemployment, underemployment, or a change in employment was directly related to caring for and managing appointments for their child with a neurodevelopmental disability. Parents also reported that leaving the workforce invoked a loss of self-identity. These findings are consistent with previous literature that found parents of children with disabilities often have higher unemployment rates, despite no difference in education level when compared to parents of children without disabilities (32, 33).

Many of the parents also reported secondary medical diagnoses and delays across developmental domains. Approximately, 30.7% of the parents reported that their child has a mental or behavioral health diagnosis. These include anxiety, depression, obsessive compulsive disorder, and oppositional defiant disorder. Hirota and King previously reported that children with ASD are more likely to have higher rates of anxiety (20% vs. 7%) and depression (11% vs. 5%) compared to children without a disability (20). However, accessing behavioral health services in Appalachia can be difficult due to the lack of providers. Precisely 50% of the children also have a speech or language delay and more than 25% of children have delays in the cognitive, learning, motor, and social emotional domains. Sanchack and Thomas recommend early and intensive behavioral therapies to improve outcomes across developmental domains (19). Parents also report that these secondary diagnoses and developmental delays can inhibit their child from making meaningful friendships or participating within their communities. This was evident in the responses from the F-Words Life Wheel, as Friendship was rated the lowest in satisfaction by parents with an average score of 5.3 out of 10.

Seven of the parent participants (41.2%) had multiple children with a neurodevelopmental disability. Five parents had two children, and two parents had three children with a neurodevelopmental disability. This is unsurprising as both ADHD and ASD are thought to have genetic etiologies (34, 35). However, managing the health and wellbeing of two (or more) children with unique needs can be very challenging for parents. Parents discussed the difficulty of managing different medical schedules, education, and behaviors of their children. They also discuss the fear of receiving a diagnosis for their younger child and contemplations about how they were going to manage their daily lives after receiving the diagnosis for their child.

To create targeted clinical interventions to improve performance across developmental domains and participation for children with neurodevelopmental disabilities living in Appalachia, or other rural communities, it is important to consider both intrinsic (personal) and extrinsic (environmental) factors. Based on the findings from our study, we have several recommendations for clinical interventions for children with disabilities living in rural and medically underserved regions. Interventions for children with neurodevelopmental disabilities in rural communities may target any developmental domain, as delays exist in each. Targeted clinical interventions within medically underserved regions should first consider the shortage of service providers to ensure the sustainability of their efforts. Group-based interventions may be considered as they reduce the number of trained professionals required, while providing care to more than 1 child at a time. Group-based interventions may also help to target the development of social emotional skills and help children with neurodevelopmental disabilities create meaningful friendships, an area parents identified as being important to their child's well-being. Interventions that utilize telehealth services may also help to improve access to specialized care by expanding the care network outside of the region. Both synchronous and asynchronous telehealth services should be explored, as the internet within rural communities are not always reliable. Finally, many parents in our study had multiple children with disabilities and expressed feelings of being overwhelmed. This is not unique to the Appalachian region and should be considered during the development of research studies targeting children with disabilities. Families of children with disabilities may need access to affordable respite care options. Future studies may investigate the use and cost of respite care to better identify gaps in this region to inform clinical interventions and policy decisions. In addition, studies that utilize parent-coaching as a model should be aware of these feelings, as it may inadvertently result in non-adherence to the intervention.

To further improve the health and well-being of these children, current policies may need to be re-examined for their effectiveness for helping children with neurodevelopmental disabilities to access needed healthcare. Existing Ohio policies and legislation do not address the gaps and barriers in healthcare that we have discussed in this paper. Many Appalachian counties are federally designated as HPSA (6, 7), meaning not enough providers to meet the healthcare needs of children living in these regions. Many rural regions of the United States face similar challenges. A study by Probst et al. in 2018 (7) found that 56.6% of rural communities lack even a single pediatrician. Specialists are even more scarce. This gap in healthcare creates barriers for families to accessing services due to required travel (8, 13–15). Telehealth has been proposed as a solution, however, Ohio and its bordering states face barriers to telehealth, because telehealth requires stable internet connection—a rarity in certain regions of Appalachia and throughout rural communities. In the North Central region in Appalachia, only 83.1% of households have access to broadband internet subscriptions. Increasing access to affordable and high-speed broadband internet within this region may help to actualize the routine use of telehealth services in this medically underserved region. Policies may consider incentivizing not only medically trained personnel, but also rehabilitation professionals to work in these rural regions to help combat the shortage of providers and provide children with the care they need to fully participate within their communities.

Barriers related to health insurance coverage remains at the forefront of the literature regarding access to healthcare in Appalachia (4, 10, 11, 15, 17, 18). Ohio Medicaid Expansion under the Affordable Care Act (ACA) significantly increased healthcare coverage for low-income individuals during the beginning of the COVID-19 pandemic. Ohio Medicaid has begun disenrolling people since 2022 impacting hundreds of thousands of Ohioans. This directly impacts a family's ability to afford healthcare for their children. Regarding disability home healthcare coverage, Ohio provides Medicaid waivers for individuals with disabilities to receive services at home or in community settings, yet access varies widely due to funding and workforce shortages in rural areas. Policies that incentivize trained community workers may help to mitigate this shortage.

Studies consistently highlight poor health outcomes in Appalachian Ohio, including higher rates of chronic illnesses and substance use. This is consistent with other Appalachian regions, which includes 13 states and millions of children. Access to healthcare is hindered by healthcare provider shortages, transportation barriers, and socioeconomic challenges, even with the previous Medicaid expansion. Both policy goals and research highlight the need to address rural healthcare provider shortages, improve service accessibility, and expand funding for critical health programs. Policies and studies acknowledge telehealth as a promising solution for rural areas but note the challenges of broadband access in Appalachia and in other rural regions. Existing policies often highlight theoretical access to services, but research reveals persistent gaps in actual utilization, especially among low-income and disabled populations. Research findings emphasize cultural and social barriers, such as stigma and mistrust of systems, which are less frequently addressed in policy design.

Children with disabilities deserve the highest level of care, regardless of where they live. The themes developed from this research study may help guide future clinical research projects and policy changes to improve the health and wellbeing of children with disabilities living in rural and low-resourced regions of the US.

Limitations: There are several limitations to this study. First it is a small sample size that lacks diversity, which limits the generalizability of the results. These findings are specific for children with neurodevelopmental disabilities living in the North Central Appalachian region. However, readers may consider this as a potential starting point for clinical research in other rural and low-resourced regions within the US.

## Data Availability

The datasets presented in this article are not readily available because these data include identifiable information. Requests to access the datasets should be directed to bican@ohio.edu.

## References

[1] Morrone M. Environmental justice and health disparities in appalachia, Ohio. In: Liotta PH Mouat DA Kepner WG Lancaster JM, editors. Environmental Change and Human Security: Recognizing and Acting on Hazard Impacts. Dordrecht: Springer Netherlands (2008). p. 299–323.

[2] Singh GK Kogan MD Slifkin RT. Widening disparities in infant mortality and life expectancy between appalachia and the rest of the United States, 1990–2013. Health Aff (Millwood). (2017) 36(8):1423–32. 10.1377/hlthaff.2016.1571 28784735

[3] Driscoll AK Ely DM. Maternal characteristics and infant outcomes in appalachia and the delta. Natl Vital Stat Rep. (2019) 68(11):1–15. 32501206

[4] Smith LH Holloman CH. Health status and access to health care services: a comparison between Ohio’s rural non-Appalachian and Appalachian families. Fam Community Health. (2011) 34(2):102–10. 10.1097/FCH.0b013e31820de961 21378506

[5] Earley E Asti L Chisolm D. Comparative analysis of health care needs among children with special health care needs in Ohio’s metropolitan and Appalachian counties. J Health Care Poor Underserved. (2015) 26(3):668–75. 10.1353/hpu.2015.0105 26320903

[6] Smith LH Holloman C. Comparing child health, access to care, and utilization of health services between Ohio appalachia’s river and non-river bordering counties. J Community Health. (2011) 36(5):819–30. 10.1007/s10900-011-9380-8 21350885

[7] Probst JC Barker JC Enders A Gardiner P. Current state of child health in rural America: how context shapes children’s health. J Rural Health. (2018) 34(Suppl 1):s3–12. 27677973 10.1111/jrh.12222 PMC5373918

[8] Maganty A Byrnes ME Hamm M Wasilko R Sabik LM Davies BJ Barriers to rural health care from the provider perspective. Rural Remote Health. (2023) 23(2):7769. 37196993 10.22605/RRH7769

[9] Buchalter RB Gentry EG Willis MA McGinley MP. Disparities in spatial access to neurological care in appalachia: a cross-sectional health services analysis. Lancet Reg Health Am. (2022) 18:100415. 36844018 10.1016/j.lana.2022.100415 PMC9950666

[10] Morrone M Cronin CE Schuller K Nicks SE. Access to health care in appalachia. J Appalach Health. (2021) 3(4):123–36. 35769826 10.13023/jah.0304.10 PMC9183790

[11] Blackburn CC Nuzhath T. An exploration of barriers to access to healthcare in hancock county, Tennessee: a qualitative study. Health Expect. (2024) 27(3):e14074. 10.1111/hex.14074 38769887 PMC11106589

[12] Fehr KK Leraas BC Littles MMD. Behavioral health needs, barriers, and parent preferences in rural pediatric primary care. J Pediatr Psychol. (2020) 45(8):910–20. 10.1093/jpepsy/jsaa057 32766670

[13] Phillippi JC Myers CR Schorn MN. Facilitators of prenatal care access in rural appalachia. Women Birth. (2014) 27(4):e28–35. 10.1016/j.wombi.2014.08.001 25181958

[14] Bush ML Hardin B Rayle C Lester C Studts CR Shinn JB. Rural barriers to early diagnosis and treatment of infant hearing loss in appalachia. Otol Neurotol. (2015) 36(1):93–8. 10.1097/MAO.0000000000000636 25325844 PMC4268139

[15] Bush ML Alexander D Noblitt B Lester C Shinn JB. Pediatric hearing healthcare in Kentucky’s Appalachian primary care setting. J Community Health. (2015) 40(4):762–8. 10.1007/s10900-015-9997-0 25672888 PMC4490996

[16] Yue JK Upadhyayula PS Avalos LN Cage TA. Pediatric traumatic brain injury in the United States: rural-urban disparities and considerations. Brain Sci. (2020) 10(3):135. 10.3390/brainsci10030135 32121176 PMC7139684

[17] Nelson BB Ratushnyak D Richards A Sabo RT Wolf ER Krist AH. Using claims data to map unmet service needs for early childhood developmental disabilities in Virginia. Acad Pediatr. (2023) 23(2):457–63. 10.1016/j.acap.2022.09.003 36108999 PMC10008751

[18] Razdan R Stevens LD Ritchie M Kennedy T Saldivar S Carr MM. Parents’ reports of barriers to care for pediatric otolaryngology patients. Int J Pediatr Otorhinolaryngol. (2019) 126:109617. 10.1016/j.ijporl.2019.109617 31398590

[19] Sanchack KE Thomas CA. Autism spectrum disorder: primary care principles. Am Fam Physician. (2016) 94(12):972–9. 28075089

[20] Hirota T King BH. Autism spectrum disorder: a review. JAMA. (2023) 329(2):157–68. 10.1001/jama.2022.23661 36625807

[21] Rajaprakash M Leppert ML. Attention-deficit/hyperactivity disorder. Pediatr Rev. (2022) 43(3):135–47. 10.1542/pir.2020-000612 35229109

[22] Thapar A Cooper M. Attention deficit hyperactivity disorder. Lancet. (2016) 387(10024):1240–50. 10.1016/S0140-6736(15)00238-X 26386541

[23] Scarpa A Jensen LS Gracanin D Ramey SL Dahiya AV Ingram LM Access to autism spectrum disorder services for rural Appalachian citizens. J Appalach Health. (2020) 2(1):25–40. 35769534 10.13023/jah.0201.04 PMC9138840

[24] Christiansen CH Bass J Baum CM. Occupational Therapy: Performance, Participation, and Well-Being. 4th ed New York: Routledge (2024). p. 692.

[25] Bass JD Marchant JK de Sam Lazaro SL Baum CM. Application of the person-environment-occupation-performance model: a scoping review. OTJR (Thorofare N J). (2024) 44(3):521–40. 38519867 10.1177/15394492241238951 PMC11180417

[26] Arozullah AM Yarnold PR Bennett CL Soltysik RC Wolf MS Ferreira RM Development and validation of a short-form, rapid estimate of adult literacy in medicine. Med Care. (2007) 45(11):1026–33. 10.1097/MLR.0b013e3180616c1b 18049342

[27] Rosenbaum P Gorter JW. The “F-words” in childhood disability: i swear this is how we should think!. Child Care Health Dev. (2012) 38(4):457–63. 10.1111/j.1365-2214.2011.01338.x 22040377

[28] Vos-Vromans DCWM Ketelaar M Gorter JW. Responsiveness of evaluative measures for children with cerebral palsy: the gross motor function measure and the pediatric evaluation of disability inventory. Disabil Rehabil. (2005) 27(20):1245–52. 10.1080/09638280500076178 16298926

[29] Dumas HM Fragala-Pinkham MA Haley SM Ni P Coster W Kramer JM Computer adaptive test performance in children with and without disabilities: prospective field study of the PEDI-CAT. Disabil Rehabil. (2012) 34(5):393–401. 10.3109/09638288.2011.607217 21988750 PMC3668545

[30] Dumas HM Fragala-Pinkham MA Rosen EL O’Brien JE. Construct validity of the pediatric evaluation of disability inventory computer adaptive test (PEDI-CAT) in children with medical complexity. Disabil Rehabil. (2017) 39(23):2446–51. 10.1080/09638288.2016.1226406 27642790

[31] Braun V Clarke V. Using thematic analysis in psychology. Qual Res Psychol. (2006) 3(2):77–101. 10.1191/1478088706qp063oa

[32] Brehaut JC Kohen DE Raina P Walter SD Russell DJ Swinton M The health of primary caregivers of children with cerebral palsy: how does it compare with that of other Canadian caregivers? Pediatrics. (2004) 114(2):e182–191. 10.1542/peds.114.2.e182 15286255

[33] Michelsen SI Flachs EM Madsen M Uldall P. Parental social consequences of having a child with cerebral palsy in Denmark. Dev Med Child Neurol. (2015) 57(8):768–75. 10.1111/dmcn.12719 26154654

[34] Genovese A Butler MG. The autism Spectrum: behavioral. Psychiatric and Genetic Associations. Genes (Basel). (2023) 14(3):677. 10.3390/genes14030677 36980949 PMC10048473

[35] Kian N Samieefar N Rezaei N. Prenatal risk factors and genetic causes of ADHD in children. World J Pediatr. (2022) 18(5):308–19. 10.1007/s12519-022-00524-6 35235183

