# Supplementary material for: Parent-identified intrinsic and extrinsic factors that influence performance across developmental domains and participation in their communities
Source: Front Pediatr. 2025 Feb 24;13:1472743. doi: 10.3389/fped.2025.1472743 (PMC11891180; doi:10.3389/fped.2025.1472743)
Supplement: Supplementary file 1 [file Table1.docx]

**Attachment 1. Interview Guide – Caregivers**

**Methodology**

These interviews will be conducted in a conversational style flowing through topics guided by the interviewer. The interview will begin with general background and overall understanding of the participant’s experience and perspective, followed by broad questions inviting the interviewee to share their input. Probe questions are used as reference points for further discussion under key topics. As each interview may be unique in how conversation will flow, these questions will be used as a guide and may not be asked in the order as written, but all key topics will be covered.

**Interview Objectives**

 Objective 1: Understand where you live and things you like to do with your child in your community

 Objective 2: Identify barriers to accessing medical or rehabilitation services

**Introduction**

Thank you for your time and willingness to participate in this interview today. My name is **[interviewer name]** and I work at Ohio University.

Before we begin, I would like to give you more information about the purpose of this research. We are conducting these interviews with parents/caregivers living in Appalachia to gain a deeper understanding of the caregiver’s experience of managing their child’s developmental delays while living in Appalachia.

This interview will last approximately 1 hour. With your permission, I would like to record the audio of this interview for note taking purposes. This recording will be destroyed after the study is completed. Names and personal identifiable information will not be reported. **<Start recording after consent is given>**

Anything you say will be kept confidential within the research team, so I encourage you to be open and honest in our conversations. Some of these questions may be sensitive in nature, such as questions about psychiatric illness. You do not have to answer any questions you do not want to, and you may end this interview at any time.

Before we begin, do you have any questions?

**General background**

This is study “Motor performance and participation in children with developmental delays living in Appalachia” and we are interviewing participant [participant ID].

Please tell me about yourself.

- How old are you?
- Where do you live?
- How would you describe the area you live in? (e.g. rural, suburban, urban)
  - Tell me a little about your neighborhood
  - What do you like to do with your child in your neighborhood?

Do you have access to parks?

Are they accessible?

Grassy spaces/trees

Access to lakes/water

- Are you currently working?
- What type of work do you do?
- What is your highest level of education?
- Are you married?
- How many children do you have?
- Who do your children live with?
- What type of insurance does your child have (e.g., private, CMH, Medicaid, etc.)
- What medical conditions does your child have?

**Understand child’s disease management**

*The following questions are about your child’s experience.*

- How long ago was your child diagnosed with [conditions]?
  - Delayed diagnosis?
- How do you and your child work together to manage their health?
- How often do you wake up to check on them at night?
- Have you ever had to take your child to the emergency room?
- What kinds of things do you do at home to make your child feel better outside of formal doctors appointments?

**Insurance**

*The following are questions about your child’s health insurance.*

- What are the pros and cons of your current health insurance coverage?
- Is there anything you would do to improve it?
- Does your child’s current insurance cover all of their medical needs?
- From a parent’s perspective, is there anything you would do to improve your child’s health insurance?
- Did you have a hard time finding doctors and other healthcare providers?
- Do you feel you have a good relationship with your child’s healthcare providers?
- Do you feel you are easily able to find a doctor or specialist to meet the needs of your child?

**Barriers to Care**

*We are also interested in learning more about what prevents you from getting the best care for your child.*

- Have you ever experienced barriers to care due to your child’s insurance coverage?
- Have you experienced barriers to care due to your distance from the hospital or other health clinics?
  - *Probes*: Pharmacy, DME, the time it takes for packages to arrive, lack of reliable transportation, cost of gas
- Have you experience any barriers to care due to the cost of healthy food that is recommended for your child’s treatment?
- Have you experienced any barriers to care due to lack of social support?
  - *Probes*: Lack of support from family, lack of support from friends, people don’t know what Have you experienced any other barriers to care? What were they?
- Now that we have discussed barriers to care, do you believe that there is anything that BCMH is doing well and that should be encouraged? If so, what?
- How far are you typically driving for an appointment? (medical and rehabilitation)
  - Get location information/address
  - What barriers do you encounter to going to medical or rehabilitation appointments?
  - How far would you be willing to go for…
    - A routine medical/rehabilitation visit
    - A specialist
    - A research study
- Do you feel like the care your child receives is adequate?

**Telehealth**

- Do you have any experience with using telehealth for medical or rehabilitation appointments?
  - If so, how would you describe this experience?
  - Probes: What would make the experience better? What were the barriers you faced during the experience?
  - Do you feel that telehealth could improve your child’s access to medical or rehabilitation care?
  - Do you feel that telehealth could improve your child’s care?

**Social Support**

Where do you receive social support and from whom?

Do you feel comfortable hiring a babysitter to watch your child or taking your child to a daycare?

Probes:

If not, what would make you feel more comfortable?

If yes, have you experienced difficulty finding secondary caregivers (babysitters, etc.) who were willing to learn about disease management?

Does your child’s grandparents or other relatives assist with your child’s welfare?

Is your child on an Individualized Education Plan?

How do you feel the plan has helped your child?

What improvements could make the plan better?

What do teachers do to help manage your child’s welfare?

Probes: School nurses?

Do you feel that they are properly trained and knowledgeable to meet the needs of your child’s health?

Does your child’s coaches or gym teachers make accommodations for your child?
