# Supplementary material for: Parent-identified intrinsic and extrinsic factors that influence performance across developmental domains and participation in their communities
Source: Front Pediatr. 2025 Feb 24;13:1472743. doi: 10.3389/fped.2025.1472743 (PMC11891180; doi:10.3389/fped.2025.1472743)
Supplement: Supplementary file 2 [file Table2.docx]

Patient Survey

*Page I*

Please complete the survey below. Thank you!

**General Family Background**

How old are you?

What is your zip code?

How would you describe the area you live in?

What is the highest level of education you have completed?

Are you currently employed?

What type of work do you do?

What is your work schedule?

Are you married or living with a long term partner?

Is your spouse/ long-term partner employed?

What is your spouse's work schedule?

What is your combined household income?

O Rural

O Suburban O Urban

O Did not graduate Highschool O GED or Highschool

O Vocational or Associates Degree O Undergraduate Degree

O Masters Degree

O Doctoral degree (MD, PhD, etc)

O Yes

O No

O Full time

O Part time

OYes

O No

OYes

O No

O Full time

O Part time

O < $50,000

O $50,000-$74,999

O $75,000-$99,999

O $100,000-$49,999

O $150,000-$199,999

O $200,000+

O Prefer not to answer

**Patient/Child Background**

What is your child's sex? O Male

O Female

What is your child's race? O Asian

O Bi-racial or Multi-racial

O Black or African American O Latino/Hispanic

O Native Hawaiian O White/Caucasian O Other

What race do you identify your child as?

Was your child born full term? OYes

O No

What was your child's gestational age at birth (weeks/days old at time of birth)?

What is your child’s date of birth?

What does your child have a delay in (ie: motor, language, cognitive skills)?

Does your child have any formal medical diagnoses?

If so, please list them.

How old was your child when they received a diagnosis?

Was this a delayed diagnosis (ie: you knew something was wrong for a long time prior to getting a diagnosis)?

Who gave your child their diagnosis?

(primary care physician, neurologist, physical medicine doctor, etc)

When did you suspect something was wrong with your child's development?

How do you and your child work together to manage their health?

How often does your child wake during a typical night?

OYes

O No

O Never or almost never

O 1-2 times

O 3-4 times

O 5+ times

What causes your child to wake up in the night?

| **How often do you take your child to get medical care in the following locations:** | | | | | | | | |
| --- | --- | --- | --- | --- | --- | --- | --- | --- |
| More than  once a week | | Once a  week | Every  other week | Once a  month | Every 2-3  months | Every 4-6  months | Twice a  year | Once a  year or less |
| Scheduled appointments with | 0 | 0 | 0 | 0 | 0 | 0 | 0 | 0 |
| physician |  |  |  |  |  |  |  |  |
| Emergency Room or Urgent Care | 0 | 0 | 0 | 0 | 0 | 0 | 0 | 0 |
| Pharmacy/Prescription pickup | 0 | 0 | 0 | 0 | 0 | 0 | 0 | 0 |
| Physical Therapy | 0 | 0 | 0 | 0 | 0 | 0 | 0 | 0 |
| Occupational Therapy | 0 | 0 | 0 | 0 | 0 | 0 | 0 | 0 |
| Speech Therapy | 0 | 0 | 0 | 0 | 0 | 0 | 0 | 0 |
| Behavioral Therapy | 0 | 0 | 0 | 0 | 0 | 0 | 0 | 0 |
| Other medical related appointments | 0 | 0 | 0 | 0 | 0 | 0 | 0 | 0 |

What other medical related appointments does your child attend?

| **Do you think the frequency of visits to the following locations is appropriate for your child?** | | | |
| --- | --- | --- | --- |
| Scheduled appointments with | Yes, the frequency is  appropriate  0 | No, we should be going LESS  o n  0 | No, we should be going MORE  o n  0 |
| physician |  |  |  |
| Emergency Room or Urgent Care | 0 | 0 | 0 |
| Pharmacy/Prescription Pickup | 0 | 0 | 0 |
| Physical Therapy | 0 | 0 | 0 |
| Occupational Therapy | 0 | 0 | 0 |
| Speech Therapy | 0 | 0 | 0 |
| Behavioral Therapy | 0 | 0 | 0 |
| Other | 0 | 0 | 0 |

Generally, how do you feel about the medical care your child currently receives?

Is there anything you would improve?

Generally, how do you feel about the rehabilitation (physical, occupational, or speech therapy) services your child is receiving?

Is there anything you would improve about your child's physical therapy services?

How often is your child participating in structured exercise including physical therapy, group fitness activities and/or a home exercise program completion?

Does your child participate in any group sports or physical activities (special olympics, horseback riding, etc)?

**Community resources and internet use**

How often is your child participating in a group sport or physical activity?

Is your child participating in the same number of sports or physical activities as they did last year?

Why are they participating in less sport or physical activities this year?

Are you active in any parent support groups?

If so, please list them.

O Less than 1 hour/week O 1-2 hours/week

O 2-3 hours/week

O >3 hours/week

OYes

0No

O One season a year O Two seasons a year

O Three seasons a year O All year

OYes

O No, they are participating in more O No, they are participating in less

OYes

0 No

**The following are questions related to insurance coverage**

What type of insurance is your child covered under?

Please specify what "other" insurance your child is covered by

Did your insurance coverage influence any medical or rehabilitation decisions for your child?

□D Private insurance (Ex: Aetna, Anthem) Medicaid

D Other Public Health insurance

□ BCMH

D Other

OYes

O No

From a parent's perspective, is there anything you would do to improve your child's health insurance?

Does your child's insurance cover all of their medical needs?

What doesn't your insurance cover?

What would you describe as "perfect health insurance" for your child?

Do you have a hard time finding doctors and other healthcare providers that are covered by your child's insurance plan?

Have you ever had to change medical providers due to limitations in health insurance coverage?

OYes

O No

O Yes

O No

O Yes

O No

Please elaborate on why you had to change medical providers related to insurance (IE: copay was too high, no coverage at all, etc)

How is your connection with your child's medical team?

How would you improve your connection with your child's medical team?

| **We are interested in learning about what challenges you face when seeking care for your**  **child. Considering the below options, please indicate if this has ever been a barrier for your family when attempting to provide the best care for your child.** | | |
| --- | --- | --- |
|  | Yes, this has been a barrier | No, this has NOT been a barrier |
| Insurance coverage | 0 | 0 |
| Distance from clinic/hospital | 0 | 0 |
| Time it takes for packages to arrive (containing medications or | 0 | 0 |
| equipment for your child) |  |  |
| Lack of reliable transportation | 0 | 0 |
| Cost of gas | 0 | 0 |
| Inability to find care for siblings or other dependents | 0 | 0 |
| Lack of equipment | 0 | 0 |
| Hours of operation of facilities | 0 | 0 |
| Personal or family work schedule | 0 | 0 |
| Lack of social support | 0 | 0 |

Please share any other barriers to care you have experienced.

**The following section relates to social support and your child's welfare**

Where do you receive social support and from whom?

Do you feel comfortable hiring a nanny or babysitter to care for your child?

What would make you more comfortable hiring a nanny/babysitter?

Have you experienced difficulty finding secondary caregivers (babysitters, etc.) who are willing to learn about disease management?

Do your child's grandparents or other relatives assist with your child's welfare?

OYes

O No

OYes

O No

OYes

O No

Please comment on how teachers and school nurses help manage your child's welfare?

Do your child's coach or gym teacher make appropriate accommodations for your child to participate?

Does your child have an IEP

Do you feel that others are properly trained and knowledgeable to meet the needs of your child's health?

OYes

O No

OYes

O No

OYes

O No

Who do you feel needs more training to properly care for your child and identify areas where knowledge is lacking?

Of the topics covered by this survey, is there

anything you would like to add more about or anything you feel should have been asked?
