# Supplementary material for: Parent-identified intrinsic and extrinsic factors that influence performance across developmental domains and participation in their communities
Source: Front Pediatr. 2025 Feb 24;13:1472743. doi: 10.3389/fped.2025.1472743 (PMC11891180; doi:10.3389/fped.2025.1472743)
Supplement: Supplementary file 3 [file Table3.docx]

Barriers in Appalachia

Codes

| Name | Description |
| --- | --- |
| Babysitters and Caregivers |  |
| Family and other relatives assist |  |
| Issues finding experienced caregivers |  |
| Other |  |
| Barriers to Care |  |
| Access to Technology |  |
| Distance from hospital and health clinics |  |
| DME Issues |  |
| Lack of reliable transportation |  |
| Other |  |
| Pharmacy Issues |  |
| Shipping Issues |  |
| Waitlist |  |
| Barriers to Healthy Food for Child's Treatment |  |
| Food availability |  |
| Food insecure |  |
| Food too expensive |  |
| No barriers |  |
| can |  |
| Connection with HCP and Staff |  |
| Connection needs improvement |  |
| Good connection |  |
| Other |  |
| Poor connection |  |
| Cons of their current health insurance coverage |  |
| High copays |  |
| Lack of coverage for specialists |  |
| Lack of options |  |
| Other |  |
| DX History |  |
| Delayed Diagnosis |  |
| Missed Diagnosed |  |
| Still undetermined |  |
| Family Dynamics |  |
| Family lives faraway |  |
| Family lives nearby |  |
| Multiple children with disabilities |  |
| One child with disabilities |  |
| Improvements for health insurance coverage |  |
| Better coverage |  |
| Lack of specialty care coverage |  |
| Other |  |
| Issues Finding Doctors and HCPs |  |
| Distance |  |
| Lack of specialists |  |
| Other |  |
| Out of network |  |
| Managing Child's Healthcare |  |
| Child and parent do not work together |  |
| Child and parent work together |  |
| Emergency Room Visits |  |
| Overnight health management |  |
| Pros of their current health insurance coverage |  |
| Affordable copays |  |
| Coverage for specialists |  |
| Options to see other specialists |  |
| Other |  |
| Social Support |  |
| Lack of support |  |
| Lack of support from friends |  |
| Strong social support |  |
| Telehealth |  |
| Barriers |  |
| Benefits |  |
